# Anti-Fatigue Properties of Tartary Buckwheat Extracts in Mice

**DOI:** 10.3390/ijms12084770

**Published:** 2011-07-25

**Authors:** Hong-Mei Jin, Ping Wei

**Affiliations:** Yiwu Industrial & Commercial College, Yiwu 322000, Zhejiang, China; E-Mail: WeiPing75@qq.com

**Keywords:** anti-fatigue, tartary buckwheat extracts, exhaustive swimming test, mice

## Abstract

Anti-fatigue properties of tartary buckwheat extracts (TBE) was investigated in male Kunming mice. The animals were divided into four groups. The first group, designated as the control group (control), was administered with distilled water by gavage every day for 28 days. The other three groups, designated as TBE treatment groups, were administered with TBE of 60, 120 and 240 mg/kg body weight, respectively, by gavage every day for 28 days. Exhaustive swimming time, blood lactic acid (BLA), blood urea nitrogen (BUN), tissue glycogen, glutathione peroxidase (GPx) and superoxide dismutase (SOD) of mice after swimming were determined. The results showed that tartary buckwheat extracts had anti-fatigue properties, which extended the exhaustive swimming time of mice, effectively inhibiting the increase of BLA, decreasing the level of BUN, increasing the tissue glycogen content and the activities of SOD and GPx of mice. However, further study is needed to elucidate the exact mechanism of the effect of TBE on fatigue.

## 1. Introduction

Fatigue is known to be accompanied by a feeling of extreme physical or mental tiredness, resulting from severe stress and hard physical or mental work [1,2]. It can be divided into two categories: physical fatigue caused by such things as forced exercise or swimming; mental fatigue caused by sleep deprivation, *etc*. [3,4]. Physical fatigue is thought to be accompanied by deterioration in performance [5–7]. There are several theories about the mechanisms of physical fatigue, including exhaustion theory, clogging theory and radical theory, *etc*. Exhaustion theory and radical theory have attracted most interest. Exhaustion theory suggests that during exercise, many energy sources, such as glucose and liver glycogen, will be exhausted, thus leading to physical fatigue [8]. Radical theory suggests that intense exercise can produce an imbalance between the body’s oxidation system and its anti-oxidation system. The accumulation of reactive free radicals will put the body in a state of oxidative stress and bring injury to the body by attacking large molecules and cell organs [7]. Several studies have shown that exogenous dietary antioxidants can decrease the contribution of exercise-induced oxidative stress and improve the animal’s physiological condition [9–12]. In the past few decades, health scholars and athletic physiologists have been looking for natural antioxidant components that can not only improve athletic ability, postpone fatigue and accelerate the elimination of fatigue in human beings, but also have few side effects [13].

The genus Fagopyrum has about 15 species distributed in different parts of the world [14]. Among these species, only two types of buckwheat are used as food around the world: common buckwheat (*Fagopyrum esculentum*) and tartary buckwheat (*Fagopyrum tataricum*) [15–17]. Common buckwheat is grown almost on all continents, and tartary buckwheat originated in eastern Tibet or northwestern Yunnan in China and is grown only in Asia, Europe, and North America. It has been reported that the general composition of crude protein, crude fiber, crude fat, and crude ash of common buckwheat and tartary buckwheat are essentially the same [16]. Moreover, tartary buckwheat may even contain more bioactive components than common buckwheat. For instance, it has been reported that the flavonoid content of tartary buckwheat is higher than that of common buckwheat [18]. Gu reported that the content of flavonoids in tartary buckwheat could be as high as 7% [19]. Li *et al.* found that the types of flavonoids in tartary buckwheat were quercitin, kmaepferol, rutin, kmapferol-3-rutinoside [20]. Previous research has demonstrated that tartary buckwheat extracts (TBE) have diverse pharmaceutical effects including antihypertensive, antioxidant, hypoglycemic and hypolipidemic [15,21–23]. Especially, TBE has higher antioxidant activities, and it has been reported as strong antioxidants, scavengers of a wide range of reactive oxygen species and inhibitors of lipid peroxidation [24–26]. The antioxidant activities of TBE might be related to these flavonoids and it is an important factor to reduce or prevent fatigue. However, little information about the anti-fatigue effects of TBE is currently known. Therefore, the present study was designed to evaluate anti-fatigue properties of TBE in an animal model for fatigue. The effects of TBE on biochemical markers for fatigue were also assessed.

## 2. Results and Discussion

### 2.1. The Effects of TBE on Exhaustive Swimming Time of Mice

**Figure 1.**
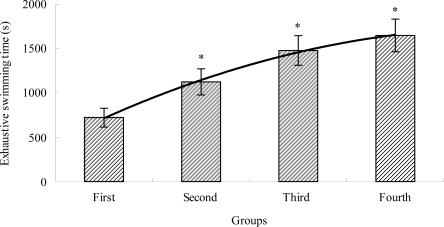
The effects of tartary buckwheat extracts (TBE) on exhaustive swimming time of mice. Values are means ± SD. * *P* < 0.05 when compared to the control group (first group).

The improvement of exercise endurance is the most powerful macro representation of anti-fatigue enhancement [27]. In the present study, we selected an exhaustive swimming test for evaluation of the extent of physical fatigue. The length of the exhaustive swimming time indicates the degree of fatigue [28]. As shown in Figure 1, the swimming time of the TBE treatment groups (second, third and fourth group) were longer (*P* < 0.05) than that of the control group (first group). The swimming time of the second, third and fourth group increased by 56.03%, 104.99% and 128.57%, respectively. Our results suggested that the different doses of TBE could elevate the exercise endurance of mice, which indicated that TBE had anti-fatigue effects.

### 2.2. The Effects of TBE on BLA of Mice

**Figure 2.**
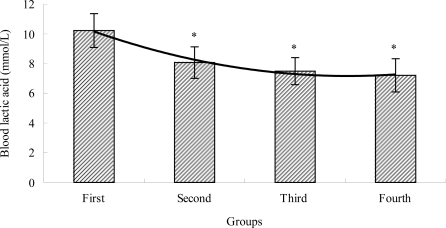
The effects of TBE on blood lactic acid of mice. Values are means ± SD. * *P* < 0.05 when compared to the control group (first group).

Blood lactic acid (BLA) is the glycolysis product of carbohydrate under anaerobic conditions, and glycolysis is the main energy source for intense exercise in a short time. The accumulation of BLA is a reason for fatigue during physical exercise, and medicine may inhibit the accumulation of BLA and accelerate the clearance of BLA, which s the anti-fatigue activity [29–31]. Therefore, BLA is one of the important indicators for judging the degree of fatigue. As shown in Figure 2, after swimming, the level of BLA in the TBE treatment groups (second, third and fourth group) were lower (*P* < 0.05) than that of the control group (first group). BLA level of the second, third and fourth group decreased by 26.77%, 36.58% and 41.89%, respectively. Our results suggested that the different doses of TBE could inhibit the increase of BLA of mice after swimming, which indicated that TBE could postpone the appearance of fatigue.

### 2.3. The Effects of TBE on BUN of Mice

**Figure 3.**
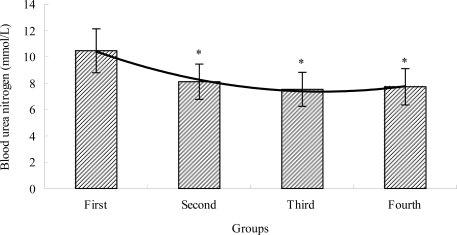
The effects of TBE on blood urea nitrogen of mice. Values are means ± SD. * *P* < 0.05 when compared to the control group (first group).

Blood urea nitrogen (BUN) is the metabolic output of protein and amino acid. Urea is formed in the liver as the end product of protein metabolism and is carried by the blood to the kidneys for excretion [32]. Protein and amino acids have a stronger catabolic metabolism when the body cannot obtain enough energy by sugar and fat catabolic metabolism, after a long time of exercise; urea nitrogen obviously increases at this time [32]. There is a positive correlation between the urea nitrogen *in vivo* and exercise tolerance. In other words, the worse the body is adapted for exercise tolerance, the more significantly the urea nitrogen level increases [33,34]. Therefore, BUN is another index of fatigue status. As shown in Figure 3, after swimming, the level of BUN in the TBE treatment groups (second, third and fourth group) were lower (*P* < 0.05) than that of the control group (first group). BUN level of the second, third and fourth group was decreased by 28.94%, 38.86% and 35.45%, respectively. Our results suggested that the different doses of TBE could decrease the level of BUN of mice after swimming, which indicated that TBE could reduce protein metabolism and ameliorates fatigue.

### 2.4. The Effects of TBE on Tissue Glycogen of Mice

Enhancement of exercise capacity could be accounted for by a reduced rate of hepatic and muscle glycogen breakdown and by a greater potential for fatty acid metabolism [35]. The determinant role of glycogen stores in the capacity for prolonged exercise has been established for many years. Liver glycogen depletion might be an important factor in the development of fatigue because as liver glycogen is depleted during exercise there is an inability to maintain blood glucose level, and the ensuing hypoglycemia could result in impaired nervous function [36]. Also, the importance of muscle glycogen levels in endurance exercise has been demonstrated and it is suggested that depletion of muscle glycogen was a factor in fatigue and exhaustion [37]. As shown in Table 1, after swimming, tissue (liver and muscle) glycogen contents of the TBE treatment groups (second, third and fourth group) were higher (*P* < 0.05) than that of the control group (first group). These data indicated that the anti-fatigue activity of TBE may be related to the improvement in the metabolic control of exercise and the activation of energy metabolism.

### 2.5. The Effects of TBE on GPx and SOD of Mice

A vast amount of evidence indicates that reactive oxygen species (ROS) are responsible for exercise-induced protein oxidation, and contribute strongly to muscle fatigue [38]. Fortunately, endogenous and exogenous antioxidant defense systems in the body can cope with ROS, including vitamin E, vitamin C, beta-carotene, and antioxidant enzymes (SOD, GPx, and catalase) [39]. SOD dismutates superoxide radicals to form H_2_O_2_ and O_2_. GPx is an enzyme responsible for reducing H_2_O_2_ or organic hydroperoxides to water and alcohol, respectively. These antioxidant defense mechanisms become weaker during chronic fatigue and other disease conditions [7,40]. Thus, the improvement in the activities of these defense mechanisms can help to fight against fatigue. As shown in Table 2, after swimming, GPx and SOD activities of muscle of the TBE treatment groups (second, third and fourth group) were lower (*P* < 0.05) than that of the control group (first group). These data indicated that TBE could promote increases in the activities of these antioxidant enzymes, again supporting that TBE has an anti-fatigue effect.

## 3. Experimental Section

### 3.1. Plant Material

The grains of tartary buckwheat for this study were purchased from Zhejiang agricultural institution (Hangzhou, China). They were grinded into powder (180 micrometer) by a domestic grinding machine (LH-08B, Jishou Zhongcheng Pharmaceutical Machinery Factory, Hunan, China) to obtain the tartary buckwheat flour.

### 3.2. Chemicals and Reagents

Standards of rutin were obtained from Sigma Co. (St. Louis, MO, USA). Assay kits for determination of blood lactic acid (BLA) were purchased from Beijing Leadman Biochemistry Technology Co. Ltd. (Beijing, China). Assay kits for determination of blood urea nitrogen (BUN), tissue glycogen, glutathione peroxidase (GPx), and superoxide dismutase (SOD) were purchased from Nanjing Jiancheng Biotechnology Institute (Nanjing, China). All other chemicals used were analytical grade.

### 3.3. Preparation of Tartary Buckwheat Extracts

Tartary buckwheat extracts (TBE) were prepared according to the method described by Cao *et al.* [15]. In brief, 10 g of tartary buckwheat flour was extracted by 200 mL ethanol-water (70:30, v/v) for 40 min using an ultrasonic generator (KQ2200E, Kunshan Ultrasonic Instrument Co. Ltd., Kunshan, China). The supernatant and the residues were separated by vacuum-filtration. The residues were extracted again with the above described method. The first and second extraction solutions were mixed and the solvent was evaporated in the case of vacuum with the rotatory evaporator and with temperature controlled at 40 °C, and the residues were frozen-dried and stored at 4 °C until use.

### 3.4. Measuring the Contents of Flavonoids

The contents of flavonoids were measured by means of UV-Vis spectrophotometry with chromogenic system of NaNO_2_-Al (NO_3_)_3_-NaOH [41–43]. In brief, 5 mL of buckwheat ethanolic extracts was mixed with 0.3 mL of NaNO_2_ (5%) solution and then 0.3 mL of Al(NO_3_)_3_ (10%) solution was added after 6 min. Next, exactly 4 mL of NaOH (1 mol/L) solution was added. The final mixture was shaken three times and the absorbance was measured at 510 nm. Rutin (50–500 mg/mL in 60 mL/100 mL ethanol) was used as a standard, giving a linear regression equation as follows: Absorbance = 0.1361 rutin (mg/mL) − 0.0738 (R^2^ = 0.9997). Flavonoids were expressed as rutin equivalents and the reaction mixture without rutin was used as the control.

### 3.5. Animals and Care

A total of ninety-six 3-month-old male Kunming mice (20 ± 1.0 g) were obtained from the Experimental Animal Center of Zhejiang Province (Certificate No. 20061372). The mice were housed in conventional cages with free access to water and rodent chow at 20–22 °C with a 12-hour light-dark cycle for seven days to allow for acclimatization before the experiments were performed. The experimental protocol was approved by the Local Animal Care Committee at Yiwu Industrial & Commercial College. All the experimental procedures were carried out in accordance with the international guidelines for Care and use of laboratory animals.

### 3.6. Grouping of Animal

The mice were randomly divided into four groups, each consisting of 24 mice. The first group designated as control group (control) was administered with distilled water by gavage every day for 28 days. The second, third and fourth group designated as BEE treatment groups were administered with BEE of 60, 120 and 240 mg/kg body weight, respectively, by gavage every day for 28 days.

### 3.7. Exhaustive Swimming Test

Exhaustive swimming test was employed in our study to evaluate the anti-fatigue effects of TBE. The procedure used was described previously [44–46]. The swimming test was carried out in an adjustable-current water pool (50 cm × 50 cm × 40 cm), filled with water to a depth of 30 cm and maintained at a temperature of 25 ± 1 °C. The current in the pool was generated by circulating water with a pump, and the strength of the current was adjusted to 8 l/min with a water flow meter (type F45500, Blue White Co., Westminster, CA, USA). The water was agitated to keep the mice limbs moving. After the final treatment with TBE or distilled water, the mice were allowed to rest for 30 min. Then, eight mice were taken out from each group for the exhaustive swimming test. A lead block (5% of body weight) was loaded on the tail root of the mice and the swimming time to exhaustion was recorded. The mice were determined to be exhausted when they failed to rise to the surface to breathe after 7 s [47].

### 3.8. Analysis of Biochemical Parameters

After the final treatment with TBE or distilled water, the mice were allowed to rest for 30 min. Then, 16 mice were taken out from each group for the biochemical parameters analysis. The mice were forced to swim for 90 min without loads [48]. Immediately after swimming exercise, the mice were anesthetized with ether, and blood samples were collected in heparinized tubes by heart puncture for the determination of BLA and BUN. Liver and gastrocnemius muscle were quickly dissected out, frozen in liquid nitrogen, and kept at −70 °C until analysis for determination of tissue glycogen, GPx and SOD. Determination and method of operation were performed according to the recommended procedures provided by the kits.

### 3.9. Statistical Analysis

Data were analyzed using SPSS 13.0 version. The results were expressed as the mean ± SD. The significance of the mean difference between the control group and each treatment group was determined by Student’s *t*-test. The level of *P* < 0.05 was used as the criterion of statistical significance.

## 4. Conclusions

The results show that TBE had anti-fatigue properties, which extended the exhaustive swimming time of mice, effectively inhibited the increase of BLA, decreasing the level of BUN, increasing the tissue glycogen contents and the activities of antioxidant enzymes of mice. The putative mechanism of anti-fatigue activity of TBE is that it can delay the increase of BLA, increase tissue glycogen reserve after exercise, which will help increase aerobic and anaerobic exercise capacity. In addition, tartary buckwheat is abundant in flavonoids and TBE has higher antioxidant activities, which as exogenous antioxidants can promote or interact with endogenous antioxidants to form a cooperative network of cellular antioxidants. However, further study is needed to elucidate the exact mechanism of the effect of TBE on fatigue.

## Figures and Tables

**Table 1.** The effects of TBE on tissue glycogen of mice. Values are means ± SD.

**Groups**	**Liver glycogen (mg/g)**	**Muscle glycogen (mg/g)**
First	7.25 ± 1.19	1.36 ± 0.21
Second	14.86 ± 2.34 *	2.04 ± 0.34 *
Third	18.73 ± 2.06 *	2.48 ± 0.27 *
Fourth	19.16 ± 3.28 *	2.97 ± 0.38 *

* *P* < 0.05 when compared to the control group (first group).

**Table 2.** The effects of TBE on glutathione peroxidase (GPx) and superoxide dismutase (SOD) of skeletal muscle of mice. Values are means ± SD.

**Groups**	**GPx (u/mg protein)**	**SOD (NU/mg protein)**
First	47.81 ± 4.16	76.38 ± 5.69
Second	58.29 ± 4.87 *	91.25 ± 6.84 *
Third	67.84 ± 5.23 *	113.68 ± 7.25 *
Fourth	78.23 ± 4.28 *	148.26 ± 6.81 *

* *P* < 0.05 when compared to the control group (first group).
